# An Inventory of European Birth Cohorts

**DOI:** 10.3390/ijerph17093071

**Published:** 2020-04-28

**Authors:** Claudia Pansieri, Chiara Pandolfini, Antonio Clavenna, Imti Choonara, Maurizio Bonati

**Affiliations:** 1 Department of Public Health, Laboratory for Mother and Child Health, Istituto di Ricerche Farmacologiche Mario Negri IRCCS, 20156 Milan, Italy; pansieri.claudia@gmail.com (C.P.); antonio.clavenna@marionegri.it (A.C.); maurizio.bonati@marionegri.it (M.B.); 2 Academic Division of Child Health, University of Nottingham School of Medicine, Derby DE22 3DT, UK; Imti.Choonara@nottingham.ac.uk

**Keywords:** birth cohort, infant, Europe, data collection, descriptive research

## Abstract

Many birth cohorts have been carried out. We performed a review of European birth cohorts to see the countries involved, provide a panorama of the current research topics and design, and, more generally, provide input for those creating collaborations and laying out guidelines aimed at unifying cohort methodologies to enable data merging and maximize knowledge acquisition. We searched PubMed and Embase for articles referring to longitudinal, prospective European birth cohorts and searched online cohort inventories. We found references to 111 birth cohorts, 45 of which began enrolment at birth. These cohorts began between 1921 and 2015 and represented 19 countries, with varying sample sizes (236 to 21,000 children). As of 5 January 2020, were still recruiting. The main areas addressed were allergic diseases (14 cohorts) and environmental exposure (f12 cohorts) and most cohorts were publicly funded. Given the large costs of running cohorts and the importance of long follow-up periods in identifying the risk factors for disorders thought to have a perinatal/early life etiology, current cohorts must be designed to answer research questions considering several aspects, from genetic ones to psychological, social, and environmental ones. Furthermore, universally recognized methodological aspects are needed to permit the comparison and merging of cohort data.

## 1. Introduction

Cohort studies collect data on a group of people in order to identify and quantify the relationship between exposure and outcome. They can be prospective or retrospective. In prospective cohort studies, the population is recruited regardless of exposure or outcome status and is followed for a set period until the diseases or outcomes of interest occur [1,2]. In retrospective cohort studies the population and its medical events or outcomes are examined by looking at the past. The limitations of this kind of study are linked to the limited control that the investigator has over data collection, increasing the risk of incomplete, inaccurate or inconsistent data [3]. Prospective cohort studies are useful as monitoring tools, and in this sense are similar to registries [4]. Registries can also be used to collect data prospectively and continuously, as in the collection of medical record data, reflecting clinical practice. Both cohorts and registries can be started at different times, based on their aims, can be used for different scopes, and can collect data at different time points.

There are different types of registries, from patient registries based on a disease or exposure, which collect data on patients with that characteristic, to those simply listing patients with specific diseases, e.g., rare diseases, but are not used for evaluating outcomes [5]. A cohort is more malleable and can be designed to identify causality between risk or exposure factors in early life and health in later life. In fact, birth cohorts, which start from pregnancy or birth and follow newborns for a period of time, often into adolescence or adulthood, are carried out especially with this aim, for example to assess the impact of environmental exposure during development and its effects on adult health. Substantial evidence about this link has been found in recent years [6], and increased attention is being placed on the prospective, longitudinal collection of data from subjects. This type of data collection permits the continuous collection of data and the study of various factors contemporaneously. These diverse factors range from those involved in nurturing care [7,8], i.e., family structure, social and physical environment, schooling, and health and nutritional behavior, to exposure to environmental toxins such as air pollution, allergens, metals, pesticides, and smoking [9]. All these factors have increasingly been acknowledged as having significant impact on adult health [10] and birth cohorts are fundamental in understanding the extent of their effects, as well as potential corrective interventions. Scientific evidence has shown how simple actions involving the reduction of exposure to risk factors or the promotion of protective factors in the first few years of life can prevent significant health problems in children and adults [6,11,12,13].

Many birth cohorts have been carried out around the world and many are currently ongoing [14,15,16]. Europe, especially Northern Europe, has been particularly active. In this context, we performed a review of European birth cohorts to analyze where they are based, the current enrolment status, their objectives, areas addressed, and age periods covered, with a focus on cohorts that started enrolment at birth and not in pregnancy. Our aim was to generate a panorama of the current birth cohorts’ research topics and design and to provide input for those creating collaborations and laying out guidelines aimed at unifying cohort methodologies to enable merging of data and maximize knowledge acquisition. We also aimed to understand how many birth cohorts address the impact of the family context (nurturing care) and the impact of the pediatricians’ care on child health and growth, in order to provide input for future cohort studies.

## 2. Materials and Methods

Between January and July 2019, we performed a narrative review of the European birth cohorts taking into consideration multiple sources. The search strategy is described in detail in Supplementary Material, Table S1. Inclusion criteria were: Birth cohorts that were based in a European country and collected longitudinal and prospective data on the babies. In order to not exclude pertinent publications, however, we chose search strategies with high specificity and low sensitivity and had to limit results via individual ascertainment.

We searched PubMed and Embase with the last update on 1 July 2019, limiting the results to the 20th of May 2019, with no restriction on past publication years. We excluded randomized controlled trials and articles focusing on vaccines or on genes or gene expression. Records found were downloaded in the Reference Manager 12 software (Thomson Research Soft, Carlsbad, CA, USA). The records were reviewed and, for each one, the name of the cohort it involved was noted. When this information was not available in the records’ abstracts, the articles were retrieved when possible. We also searched online birth cohort inventories to see if any additional cohorts could be found. In particular, we consulted the web-based database (http://www.birthcohorts.net), created as part of the Children Geno Network (a European FP5 Research Program) in 2005, and improved and redesigned within the European FP7 Program CHICOS project (http://www.chicosproject.eu). We also searched the cohorts listed by two EU funded research projects: The ENRIECO project [17] and the EUCCONET Network [18].

Exclusion criteria were: Vaccine studies, case-control studies designed within existing cohorts, studies that applied gene analysis or other criteria in sample selection, or cohort studies focusing only on the parents or on pregnancy outcomes, that were exclusively retrospective, that collected data from registries, or that did not involve a follow-up.

The European definition used was the UN definition [19].

We performed more detailed analyses on the subgroup of cohorts that began recruitment at birth and not during pregnancy. Cohorts that began collecting data after a few months of birth, even though patients were enrolled at birth, were included. For the more detailed analyses it was often necessary to search for additional scientific publications resulting from the single cohorts, in addition to the cohorts’ websites, in order to limit the amount of missing data. 

Two authors (Claudia Pansieri and Chiara Pandolfini) worked on different parts of the data extraction process as well as on certain overlapping parts, and all cases of uncertainty, discrepancy, or missing data were resolved through discussion, searches for additional data sources, and consensus. 

The type of funding received by the cohorts was classified into four types: Public (ministries of health, hospitals, including university hospitals, etc.), Foundation, University, and Industry.

## 3. Results

### 3.1. Identification of the Cohorts

**Figure 1:**
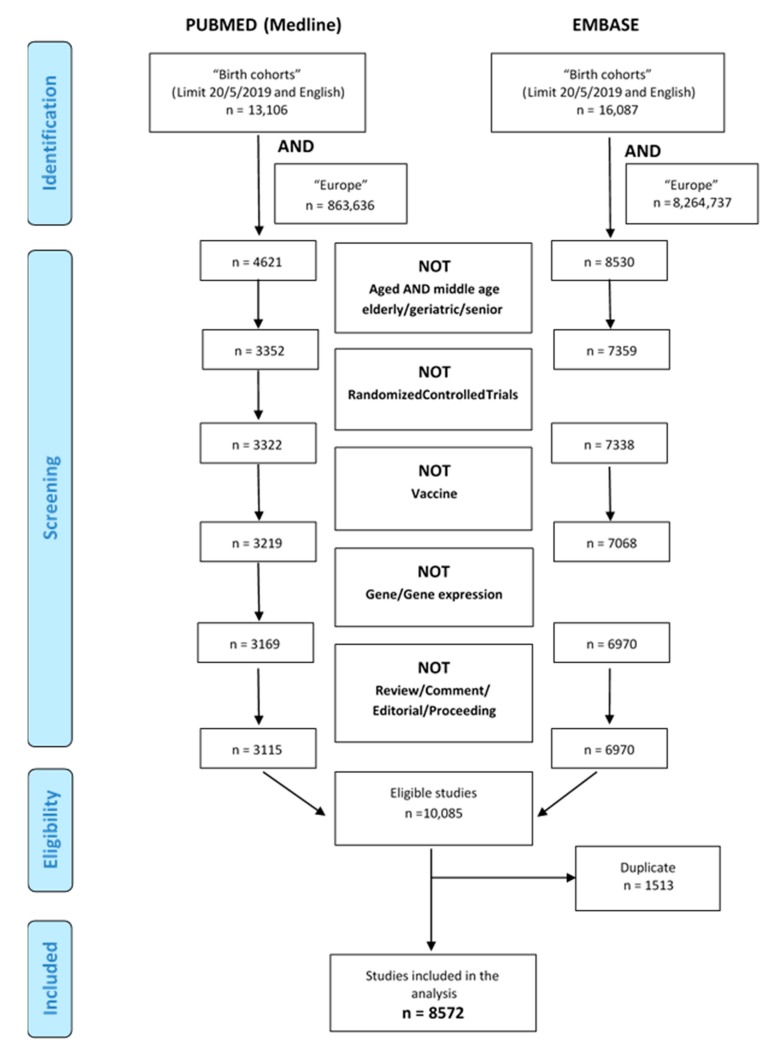
Literature selection from the two databases: Medline and Embase.

**Figure 2:**
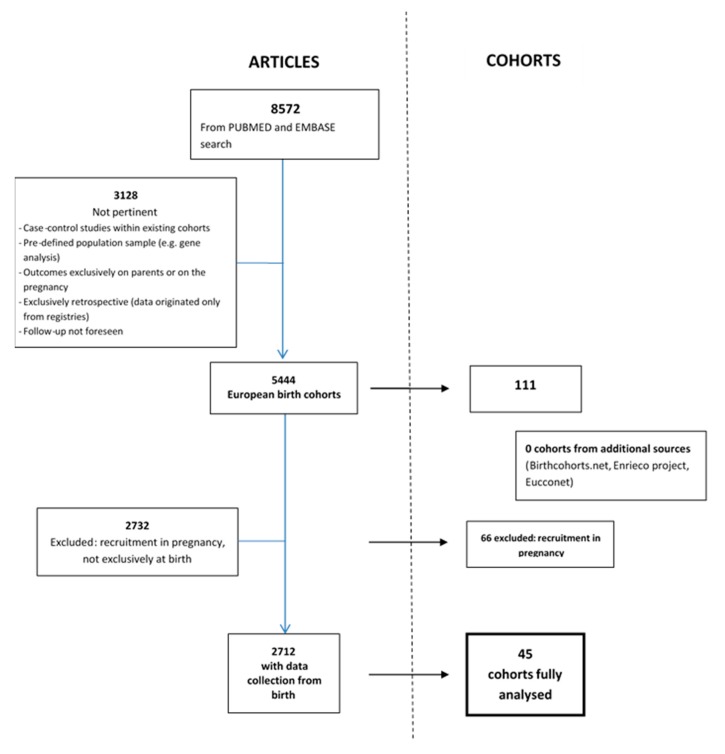
Selection of articles and number of related cohorts.

A total of 8572 articles were found through the internet-based bibliographic literature databases consulted, after exclusion of duplicates as illustrated in the PRISMA flow diagram (Figure 1). Of these, 5444 articles referred to 111 birth cohorts, while 3128 articles were not pertinent mostly because they referred to case-control studies and retrospective studies (Figure 2). The large proportion of non-pertinent articles, due to the fact that no specific indexed term exists in Medline or Embase for birth cohorts, led to the need for individual assessment of a large portion of abstracts or full-texts. Other cohorts, such as NCCGP North Cumbria Community Project [20], were also excluded because of lack of basic information such as the enrolment period and number of patients included or expected and the consequent lack of any useful information. When the online birth cohort databases were searched for additional European cohorts, none were found.

A total of 111 European birth cohorts were identified. Of these, 66 began enrolment in pregnancy (2 of which in pre-pregnancy) and 45 at birth or shortly afterwards. References of articles referring to the 45 cohorts found are listed in Supplementary Material, Table S2.

### 3.2. The European Panorama

The 111 European cohorts represented 27 different countries, including three countries represented only in the four multinational cohorts (Austria, Iceland, and Slovenia). The countries most commonly involved, in 16 cohorts each, were Germany and the UK, followed by the Netherlands (15). The number of children recruited in the different cohorts ranged from 107 to 10.8500 (median 1924). The starting year of enrolment in the different cohorts ranged from 1921 to 2016 (median 2002) and the duration of enrolment, excluding 10 with currently ongoing enrolment, and one with missing data, ranged from 1 to 23 years (median 2) (rounded to whole years). Concerning the follow-ups, 62 have ongoing follow-ups, of which 22 are lifelong and the rest of which have a duration of 1 to 31 years. The median could not be calculated because of the general nature of the description of follow-up duration for several cohorts (e.g., young adulthood).

### 3.3. The 45 Cohorts Starting Recruitment at Birth

**Figure 3:**
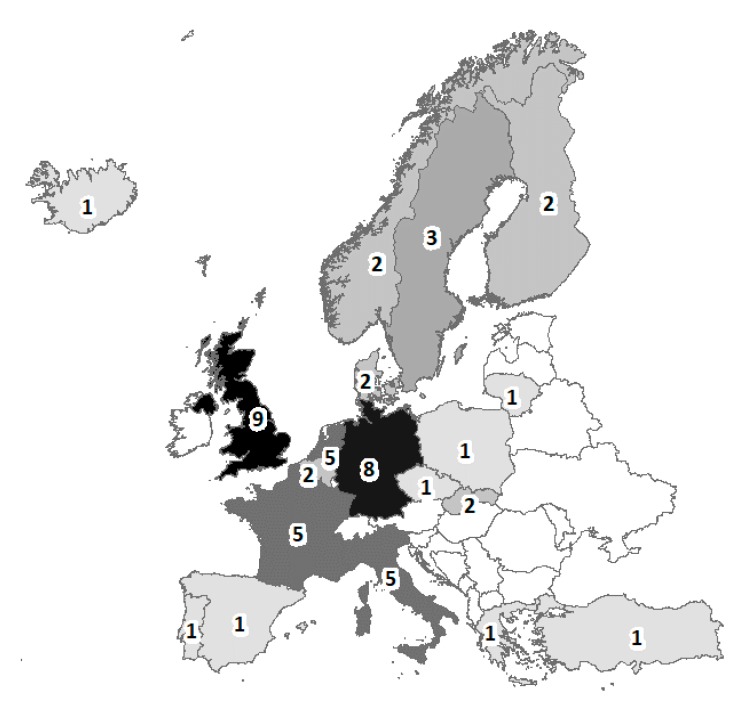
Location of the countries. The numbers refer to the number of times each country is represented in the 45 cohorts (the total is > 45 because of the multinational cohort, Europrevall). The darker the shading, the higher the numbers.

When only the subset of cohorts that began recruitment at birth was selected, 45 cohorts were present (Table 1), representing 19 European countries, 7 (37%) of which are located in Northern Europe, and 11 (58%) in Northern or Western Europe (Figure 3). Only the Europrevall cohort was multinational and involved 9 countries.

The starting years of these cohorts ranged from 1921 to 2015 (median 2002). More than half of the cohorts began in 2000 or later and 8 after 2010. The sample size of each cohort varied considerably, from 236 of the 1990 Dutch cohort to more than 21000 children of the TEDS—Twins early development study, with a mean of 4230 (median 2515). The two largest birth cohorts are located in the UK (TEDS-Twins early development study, with 21,000 children enrolled) and in FRANCE (ELFE- Etude Longitudinale Francaise depuis l’Enfance, with 18,326 children enrolled).

**Figure 4:**
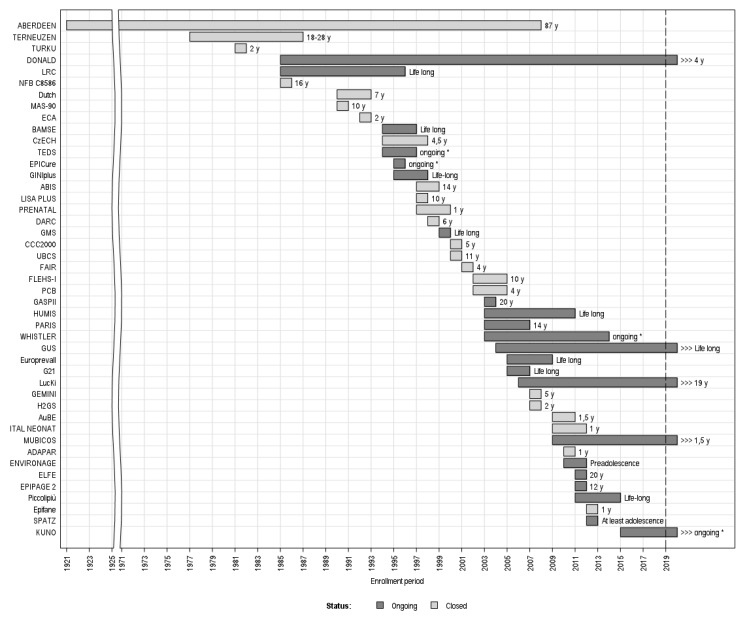
The 45 cohorts and their enrolment period, follow-up status (Ongoing/Closed), and duration (years). X-axis: enrollment period; y-axis: cohorts’ names/acronyms. * No specific follow-up termination date.; y-years.

The oldest of the 45 cohorts enrolled participants in 1921 (the 1921 Aberdeen Birth Cohort) and the youngest began enrolment in 2015 (the German KUNO-Kids birth cohort) (Figure 4). As of January 2020, the majority of cohorts were closed to recruitment since they completed enrolment of child participants. Five cohorts are currently still recruiting: DONALD (begun in 1985), GUS—Growing Up in Scotland (2004), KUNO-Kids (2015), the LucKi birth cohort (2006), and MUBICOS (2009).

Concerning the follow-up, 49% (22/45) of the cohorts are still undergoing follow-up, while the rest are definitively closed. Concerning the more recent cohorts, fifteen of the 26 (58%) cohorts set up from 2000 on, and 6 of the 8 (75%) from 2010 on, are currently ongoing. The duration of the follow-ups ranged from 1 year to life-long (Figure 4).

**Figure 5:**
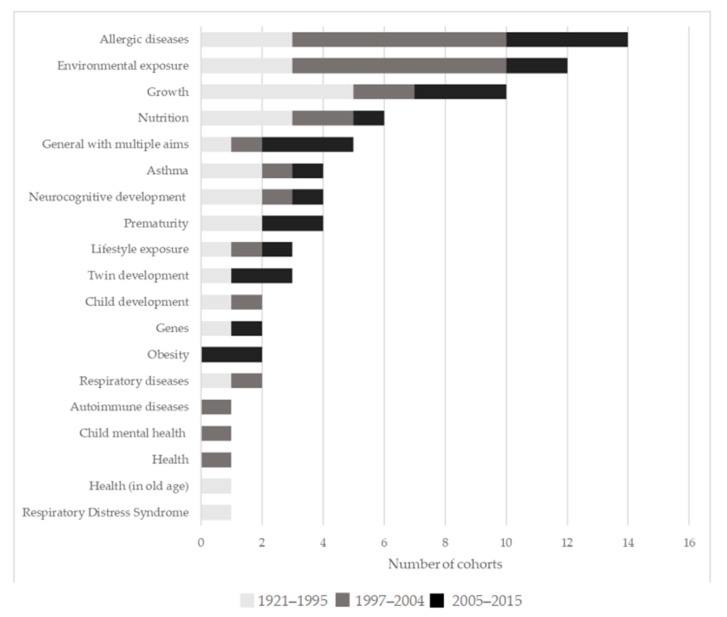
Frequency of scientific areas addressed by the cohorts, divided into three groups based on starting year of the cohort.

The aims behind the creation of the cohorts are various and cover a broad range of aspects of child health. The most frequently studied individual topics included: Allergic diseases (14 cohorts), environmental exposure (12), and growth (intended as physical growth, 10), although several cohorts (27) addressed multiple areas and were designed to test a wide range of hypotheses (Figure 5). Allergic diseases were most often studied in terms of their association with environmental exposures and asthma, but also with autoimmune diseases, lifestyle exposure, nutrition, and obesity. Environmental exposure was also studied together with genes, lifestyle exposure, neurocognitive development, and twin development, but also with asthma, autoimmune disease, growth, and nutrition. Growth was also studied together with nutrition, but also with health, neurocognitive development, and prematurity.

When divided into three groups based on age of the cohorts to see if, over time, the priorities studied changed, allergic diseases and environmental exposure were more recent priorities. Both were initially studied to a limited extent. Allergic diseases resulted as a priority area among the cohorts for the first time in 1990 and environmental exposure in 1992. More specifically, in the 1921–1995 period, growth (5 cohorts) and allergic diseases, environmental exposure, and nutrition (3 each) were the most commonly addressed areas, between 1997–2004, they were, allergic diseases and environmental exposure (7 cohorts each), and in 2005–2015 they were allergic diseases (4), and growth and general areas with multiple aims (3 each). Table 2 lists the specific objectives of the 45 cohorts.

Only three cohorts addressed the impact of the family context (nurturing care) to a certain extent among their goals, the ELFE (Etude Longitudinale Française depuis l’Enfance), Epifane, and GUS (Growing up in Scotland) cohorts. All three of these cohorts were relatively recent (2011, 2012, and 2004, respectively).

## 4. Discussion

In the review presented in this article we provide up to date information on birth cohorts in Europe with a focus on those that began data collection at birth. 

The fact that more than half of the cohorts began in 2000 or later and that many are still ongoing in terms of follow-up of participants suggests that there is a current, active interest in newborns, although with the involvement of only 9 countries, and with different aims. 

The number of participants included varied largely, although the average was of only just over 4000. With larger sample sizes, aided by the use of standard measures in the pooling of cohorts, and the joining of data from large epidemiological studies from other countries, it is possible to understand the epidemiology of diseases [21]. A long follow-up period is fundamental to assess the impact of different factors on adult health and to be able to identify possible corrective interventions. A powerful limiting factor in setting up, and running, large cohorts over large periods of time is the cost [22]. Two very large studies in the UK and US have, in fact, recently been cancelled also due to budgetary issues [23,24].

More than a third of the cohorts were established in northern Europe, where this kind of study has a long-lasting tradition (Figure 3). Health surveillance (perinatal and not) in this area of the world is often of high quality also because of the use of record linkage between health, civil, and administrative data [25]. Two thirds of the cohorts were established in Northern or Western Europe, and in high income countries in all except one case. These data are similar to those of Larsen et al., which included pregnancy cohorts as well, and whose cohorts were limited to those of greater entity and limited to 2013 [16].

Unlike the work carried out by Larsen and colleagues [16], we limited the analysis to European cohorts starting enrolment of the babies after birth. This was done because we wished to focus our study on child development in general and on the impact of nurturing care. We, however, reported in the data the cases in which the cohorts included retrospective pregnancy data.

The main areas addressed by the cohorts were allergic diseases and environmental exposure, both of which have become priority study areas more recently. The numerous cohorts addressing environmental exposure reflect increasing attention to the negative effects of pollution on health. Growth was studied more by the older cohorts, while obesity is a new research area, although all of the areas currently remain topics of interest for research, expansion of knowledge, and appropriateness of interventions. Many cohorts were designed to test a wide range of hypotheses, such as the Spatz cohort [26]. This approach addresses the identification of many risk factors for disorders thought to have a perinatal/early life etiology such as birth defects, respiratory conditions, and childhood cancer [27,28].

Other cohorts were more focused on specific topics, such as respiratory diseases (e.g., the LRC cohort) [29]. The exposure to a pattern of adverse early-life stressors, in specific age windows, influence health throughout the life cycle. The scientific evidence currently available clearly shows how even events occurring shortly after conception and up until the time a baby is delivered may lead to diseases and morbid events. These may be either present at birth or may manifest themselves later in life, in early childhood and or in adult age [11,12,30,31]. Several stressors have already been identified through the exploration of data from historical birth cohorts [32]. The early-life stressors that recently reached scientific attention are: Socioeconomic circumstances, migration, urban environment as well as lifestyle-related determinants [33].

The research results show that few cohorts have followed in detail child development as well as neurodevelopment. In general, child health is a product of biological factors and diverse sets of environmental influences, including intrauterine and social ones [10,11,12,13,14,15,34]. This implies that high-quality measures of multiple dimensions of both sets of influences need to be taken during appropriate developmental periods. Epigenetic and phenotypic measures and their associations with health outcome since conception and/or birth are increasing aims of prospective cohort studies [35]. The collection of biological samples, conducted by the majority of the ongoing cohorts, has increasingly become part of routine data collection [36] given its importance in studying the biological mechanisms of disease, and also permitting the measuring of biomarkers of environmental exposures. Biological samples, in fact, allow researchers to study how social and environmental factors leave biological imprints, independent of, or in combination with, genetic background [37].

The cohorts were supported mostly by public funds. Setting up and running cohorts, especially over long periods of time, is very important, but is also extremely costly. More economic support would be useful for setting up cohorts in all countries, and for making it possible to collect enough information, and in a suitable format, to make the cohorts comparable enough to merge their data with that of other cohorts. The industry had a limited presence in the cohorts described in this review.

The limited funds available for running cohorts inevitably influence the type of data collection employed. While most of the cohorts collected data via predefined questionnaires and face to face interviews, which are less costly, patient visits involving clinical assessment were carried out in just over half the cohorts. The Nordic countries often draw patient data from different inter-related registries, facilitating collection of also clinical data, and reducing costs [22,38]. The cohorts also used hospital records to obtain data on the mothers, the pregnancies, and the births, facilitating the collection of sufficient data from which to calculate correlations with subsequent events. The use of web-based questionnaires in assessing perinatal outcomes has also been found to be a valid way to collect data, while limiting costs [39].

The lack of commonly acknowledged guidelines on the use of common measures for data collection, along with the various data sources used by cohorts, lead to the extreme difficulty in merging or comparing data from different cohorts, a process that would permit more far-reaching, significant conclusions from the research. This is a well-recognized issue and different groups are working to address it [40].

Few cohorts also focused on family context (nurturing care) and its impact among their research areas. The family context is a fundamental issue [7] and should be a priority study area, also considering the large inequalities present between different families and the vast influence such different contexts may have on later life. The cohorts that at least partly investigated the family context were relatively recent and were set up around the years when the Lancet series addressing the evidence linking early childhood development with adult health and wellbeing began [41].

Few cohorts involved the general pediatrician (or the general practitioner) as the person delegated to collect data, highlighting the fact that primary care is a neglected resource for research [42]. With their clinical practice, pediatricians are most in contact with patients and can promote study and action. Pediatricians can play a role both in the education of parents and other caregivers, and in the implementation of curative, preventive, and health-promoting interventions through their professional practice. They can work together with other professionals in the development and execution of research with special attention to child growth and development, child mental health, and, in general, to the well-being of the future generations.

A lot of work must be done, however, to determine why some babies go on to develop disease, while others remain healthy. It is in this context that the pediatrician, or the general practitioner taking care of the newborn, play a fundamental role in describing the importance to the families of specific actions to be taken to guarantee a child’s health [43]. In such a context, the participation of general practitioners in birth cohort studies can be seen as a combative initiative, sharing and comparing their practice over time and monitoring the development of their patients.

Our aim was to describe the birth cohorts’ research topics and design, to understand their interest in the impact of the family context (nurturing care) and the general pediatricians’ role in child care and data acquisition, and to provide input for future cohort studies and for those working towards universally acknowledged guidelines for unifying cohort methodologies in order to enable data merging and the consequent maximum acquisition of knowledge. The results of this study show that a limited number of countries participates in multinational birth cohort studies and that adequate, universally recognized methodological aspects (e.g., sample size, data collected, and follow-up duration), and common health priorities, are needed in order to permit the comparison and merging of cohort data. Such an expanded amount of comparable data would permit researchers to draw more solid conclusions and stakeholders to implement the knowledge in initiatives aimed at improving people’s health.

To our knowledge, this is the first inventory of birth cohorts, both at the European and worldwide level, starting recruitment after birth. Considering pregnancy and birth cohorts together, inventories have been produced in Canada [44] and Asia [45]. Several collaborations addressing specific research questions including several worldwide birth cohorts, however, were set up in the last few years, such as the Environment and Child Health International Birth Cohort Group (ECHIBCG) [46] and the CODATwins Project [47]. The only indispensable tool that can easily be searched and that accepts registration from pregnancy and birth cohorts established all over the world is www.birthcohorts.net.

Potential limits of this study exist. It is possible that we did not identify all the European birth cohorts, but we attempted to use the most rigorous and extensive search strategy for identifying the cohorts, so we expect that a potential percentage gap would be small. This review is of a descriptive nature; we did not contact the principal investigators of the cohorts, but searched for information only via web, and this may have limited the completeness of data or led to partial data, since data found in one publication may be different from those in other publications referring to the same cohort. Furthermore, classifying the cohorts’ aims into individual scientific areas was difficult given the overlap between areas (e.g., lifestyle and environmental exposures), but the distinction was useful in order to provide a general description of the cohorts and to show their differences. For example, the four remaining cohorts labelled as addressing general areas with multiple aims were not classifiable because their aims were so widespread.

The strengths of this study are that it reports on a large number of active initiatives whose role is to look ahead, starting from birth, to monitor the development of European newborns. The findings of these cohort studies can be useful for stakeholders in allocating resources towards appropriate endeavors in order to work towards improving the health of citizens from birth.

## 5. Conclusions

The continuing follow-up of existing cohorts is the most efficient way to detect areas of improvement and windows of collaboration. Longitudinal data investments need to be directed at capturing the circumstances of tomorrow’s children and adults, i.e., current cohorts must be able to answer upcoming research questions considering several aspects: genetic and biological, psychological/social environments, medical care and medications, and lifestyle and environmental parameters. In this regard, new cohorts are periodically being set up to address the more pressing issues, such as child health and pollution [48]. We also believe that primary care should be supported, exploited and valued in public health research. Future studies should involve close collaboration with family pediatricians, or physicians caring for children, since in this new vision their role will no longer be limited to treatment of diseases, but will involve the global assistance of the child and family. The present study reveals the involvement of only a few countries. In the near future more countries should be involved in multinational birth cohort studies, with adequate, universally recognized methodological aspects (e.g., sample size, data collected, and follow-up duration), with common health priorities. The role of the European commission, in addition to supporting the setup of such multinational cohorts, is to promote, and eventually require, the implementation of commonly acknowledged parameters to allow for comparison of cohorts and data merging in order to maximize the acquisition of knowledge from such studies.

## Figures and Tables

**Table 1:** The 45 European birth cohorts analyzed.

Nation	Acronym	N. Children	Enrolment Start	Follow-Up Status	Data Collected (Q: Questionnaire, PV: Patient Visit)	Data Origin ^1^	Genetic Analysis	Biological Samples (If Taken)
Belgium	ENVIRONAGE	1080	2010	Ongoing	Q, PV	SR	Yes	Cord blood
Belgium	FLEHS-I	1196	2002	Closed	Q, PV	SR	Yes	Blood, Cord blood, Exhaled breath condensate, Meconium, Saliva
Czech Republic	CzECH	7577	1994	Closed	Q, PV	SR/PED	Yes	Cord blood, Urine
Denmark	CCC2000	6090	2000	Closed	PV	PED	No	
Denmark	DARC	562	1998	Closed	Q, PV	SR/GYN-HCP/PED	No	Blood
Finland	NFB C8586	9479	1985	Closed	Q	SR	No	Info missing
Finland	TURKU	5356	1981	Closed	PV	GYN-HCP/PED	No	
France	AuBE	302	2009	Closed	Q	SR	No	Colostrum
France	ELFE	18,326	2011	Ongoing	Q	SR	No	Cord blood, Hair, Urine
France	Epifane	3368	2012	Closed	Q	SR	No	
France	EPIPAGE 2	5567	2011	Ongoing	Q, PV	SR/GYN-HCP	No	
France	PARIS	3840	2003	Ongoing	Q, PV	SR/PED	No	Serum
Germany	DONALD	1300	1985	Ongoing	Q, PV	PED	No	Urine
Germany	GINIplus	5991	1995	Ongoing	Q, PV	PED	Yes	Cord blood, Serum
Germany	KUNO	2515	2015	Ongoing	Q, PV	SR/PED	Yes	Buccal swab, Cord blood, Gingival smears, Hair, Skin swab, Stool, Urine
Germany	LISA PLUS	3097	1997	Closed	Q	-	Yes	Serum
Germany	MAS-90	1314	1990	Closed	Q, PV	GYN-HCP/PED	Yes	Blood, Cord blood, Urine
Germany	SPATZ	1006	2012	Ongoing	Q	SR	No	Blood, Breast milk, Hair, Urine
Germany	UBCS	1022	2000	Closed	Q	SR	Yes	Breath test, Serum
Italy	ITAL NEONAT	697	2009	Closed	Q, PV	PED	No	Info missing
Italy	GASPII	708	2003	Ongoing	Q, PV	PED	Yes	Blood, Cord blood, Serum
Italy	MUBICOS	800	2009	Ongoing	Q	SR	No	Saliva
Italy	Piccolipiù	3328	2011	Ongoing	Q, PV	SR/PED	Yes	Blood, Cord blood, Urine
Multinational	Europrevall	12.049	2005	Ongoing	Q	SR	Yes	Blood, Cord blood,
Norway	ECA	3754	1992	Closed	Q, PV	SR/PED	Yes	Cord blood
Norway	HUMIS	2000	2003	Ongoing	Q	SR	No	Breast milk, Cord blood
Portugal	G21	8647	2005	Ongoing	Q	-	No	Cord blood, Serum
Slovakia	PCB	1134	2002	Closed	Q, PV	SR/GYN-HCP/PED	No	Cord blood
Slovakia	PRENATAL	1990	1997	Closed	Q, PV	GYN-HCP	Yes	
Sweden	ABIS	16,058	1997	Closed	Q, PV	PED	Yes	Blood, Breast milk, Serum
Sweden	BAMSE	4089	1994	Ongoing	Q	SR	Yes	Blood, Plasma, Urine
Sweden	H2GS	2026	2007	Closed	Q, PV	SR/PED	No	
The Netherlands	Dutch	236	1990	Closed	Q, PV	SR/GYN-HCP/PED	No	
The Netherlands	LucKi	5000	2006	Ongoing	Q, PV	SR/GYN-HCP	No	Meconium
The Netherlands	TERNEUZEN	2604	1977	Closed	Q, PV	-	No	
The Netherlands	WHISTLER	2923	2003	Ongoing	Q	-	No	
Turkey	ADAPAR	1377	2010	Closed	Q, PV	SR/PED	No	Cord blood, Serum
UK	ABERDEEN	668	1921	Closed	Q, PV	GYN-HCP	No	
UK	EPICure	308	1995	Ongoing	Q	-	No	
UK	FAIR	969	2001	Closed	Q	SR	No	
UK	GEMINI	2402	2007	Closed	Q	SR	No	
UK	GMS	1029	1999	Ongoing	Q, PV	SR	Yes	Blood, Saliva
UK	GUS	5217	2004	Ongoing	Q	SR	No	
UK	LRC	10.350	1985	Ongoing	Q	SR	No	Saliva
UK	TEDS	21.000	1994	Ongoing	Q	SR	Yes	*Info missing*

^1^ SR: Self-reported questionnaire by parent; GYN-HCP: Medical information directly from gynecologists or other health care practitioner, not pediatricians; PED: Medical information directly from pediatricians/hospital-based pediatricians.

**Table 2:** Objectives of the 45 cohorts.

Nation	Acronym	Follow-Up Status	Main Objective
United Kingdom	ABERDEEN	Closed	To examine the effects of childhood mental ability on survival up to 76 years.
Sweden	ABIS	Closed	To study the influence of environmental and genetic factors on the development of Type 1 Diabetes and other immune-mediated diseases such as allergy, asthma, coeliac disease, rheumatoid arthritis, inflammatory bowel disease, and cancer.
Turkey	ADAPAR	Closed	To establish the prevalence of atopic dermatitis and identify associated risk factors by following infants from birth until 1 year of age.
France	AuBE	Closed	To define the autonomic nervous system maturity profile obtained during the first two years of life by repeated polysomnography and 24-h electrocardiogram recordings and to determine the potential influence of this autonomic profile on sleep disorders and on cognitive development at the age of 3, as well as their impact on psychometric development at three years of age among term and preterm newborns.
Denmark	CCC2000	Closed	To study child psychopathology in the preschool and school years.
Czech Republic	CzECH	Closed	To examine rates of lower respiratory illnesses in preschool children in relation to ambient particles and hydrocarbons.
Denmark	DARC	Closed	To describe the prevalences and risk factors of sensitization and allergic diseases in childhood.
The Netherlands	Dutch	Closed	To assess the association between body mass index and cognitive ability of young children.
Norway	ECA	Closed	To determine any (detectable) association between environmental factors and the development of early childhood asthma.
France	Epifane	Closed	To describe the ages for introduction of complementary food (ICF) during the first year of life and to identify maternal and infant factors associated with practices according to their agreement with the recommendations.
United Kingdom	FAIR	Closed	To determine the incidence of parentally reported food hypersensitivity and objectively diagnosed food hypersensitivity during the first year of life.
Belgium	FLEHS-I	Closed	To study the impact of outliers, determine the optimal unit for fat-soluble biomarkers in serum and quantify the major determinants for biomarkers of exposure to polychlorinated aromatic hydrocarbons (PCAHs) in three age groups.
United Kingdom	GEMINI	Closed	To assess genetic and environmental influences in twins on growth during the first 5 y of life.
Sweden	H2GS	Closed	To increase our understanding of the concept of child health and growth from a parental perspective, focusing on parental needs, and a medical/social perspective, elucidating risk factors for growth disturbances.
Italy	ITAL NEONAT	Closed	To evaluate the role of prenatal, perinatal and postnatal conditions in determining the risk of hospitalization for bronchiolitis in a large cohort of preterms with gestational age 33 weeks or more and full-term newborns.
Germany	LISA PLUS	Closed	To examine the impact of lifestyle-related factors, air pollution and genetics on immune system and childhood allergy development.
Germany	MAS-90	Closed	To trace the development and risk factors of allergic diseases prospectively from birth, determining the influence of life-style factors, environmental exposures, and health-related behavior.
Finland	NFB C8586	Closed	To examine risk factors involved in pre-term birth and intrauterine growth retardation, and the consequences of these early adverse outcomes on subsequent morbidity.
Slovakia	PCB	Closed	To better understand the role of PCBs on children’s neurobehavioral and immunologic development.
Slovakia	PRENATAL	Closed	To assess the contribution of modifiable environmental and dietary exposures to the development of infantile atopic eczema.
The Netherlands	TERNEUZEN	Closed	To assess the relative contribution of body mass index SDS (standard deviation) changes between 0–18 y of age to adult overweight, and to identify the earliest relevant, critical growth period for adult overweight.
Finland	TURKU	Closed	To assess growth and development at two years following respiratory distress syndrome.
Germany	UBCS	Closed	To evaluate the role of maternal and perinatal factors on growth, BMI, health status of children.
Sweden	BAMSE	ongoing	To study risk factors for asthma, allergic diseases and lung function and to study factors of importance for prognosis at already established disease.
Germany	DONALD	ongoing	To evaluate the complex interrelations between nutritional behavior, food consumption, growth, development, nutritional and endocrine status, individuality, metabolism and health in children from infancy to adolescence and early adulthood.
France	ELFE	ongoing	To understand how children’s health, development and socialization are affected by their environment, family circle, schooling and living conditions.
Belgium	ENVIRONAGE	ongoing	To study the association between environmental and lifestyle factors and molecular targets of ageing measured at birth and in childhood, including telomere length and mitochondrial function
United Kingdom	EPICure	ongoing	To study the survival and complications of premature infants up until discharge from hospital.
France	EPIPAGE 2	ongoing	To examine short- and long-term outcomes of very preterm children and their determinants.
Multicenter	Europrevall	ongoing	To examine the complex interactions between food intake and metabolism, immune system, genetic background and socioeconomic factors to identify key risk factors and develop common European databases.
Portugal	G21	ongoing	To study determinants of differences in fetal and early life growth and body composition.
Italy	GASPII	ongoing	To study the risk factors for childhood diseases and deepen the insight into the interaction between the genetic and the biological/environmental component on a cohort of infants.
Germany	GINIplus	ongoing	To investigate whether the development of allergic diseases can be influenced by early childhood nutrition and to observe the natural course of atopic diseases (asthma, hay fever, neurodermatitis). Additional topics such as mental health, lung function, and nutrition were subsequently added.
United Kingdom	GMS	ongoing	To investigate the antecedents of weight faltering and to study how children grow and develop and what contributes to their health.
United Kingdom	GUS	ongoing	To understand child development through a holistic approach (social, genetic and environmental).
Norway	HUMIS	ongoing	To study microbial, persistent organic pollutants, and other environmental exposures and child health outcomes.
Germany	KUNO	ongoing	To contribute to the understanding of current child health using novel omics technologies in a systems medicine approach, to identify novel modifiable factors of child health and opportunities for prevention, and to provide a platform for investigating the feasibility and effectiveness of targeted interventions.
United Kingdom	LRC	ongoing	To study the childhood epidemiology and potential risk factors of wheezing disorders and other common respiratory problems and to determine how many disease phenotypes exist.
The Netherlands	LucKi	ongoing	To examine etiology and prognosis of atopic disease and overweight/obesity.
Italy	MUBICOS	ongoing	To study the heritability and gene-environment interaction for major neonatal and pediatric outcomes in twins.
France	PARIS	ongoing	To determine the incidence of respiratory and allergic symptoms and investigate the associations between these disorders and behavioral/environmental factors, especially indoor and outdoor pollution.
Italy	Piccolipiù	ongoing	To investigate the association between genetic, obstetric, socioeconomic, environmental and lifestyle characteristics and risk factors and infant and childhood morbidity and development, and to describe the complex interactions between genetic, epigenetic, lifestyle factors and the environment.
Germany	SPATZ	ongoing	To identify factors in childhood that influence asthma, allergies and obesity or precursors of these diseases in later life.
United Kingdom	TEDS	ongoing	To better understand how genes and the environment influence learning abilities, cognitive abilities, and behavior.
The Netherlands	WHISTLER	ongoing	To investigate determinants for wheezing illnesses (including neonatal lung function, viral infections, asthma-susceptibility genes and endotoxin exposure).

## Supplementary Materials

The following are available online at https://www.mdpi.com/1660-4601/17/9/3071/s1, Table S1: Embase and PubMed search strategies, Table S2: References of the 45 cohorts.
